# Frequency of CD19^+^CD24^hi^CD38^hi^ regulatory B cells is decreased in peripheral blood and synovial fluid of patients with juvenile idiopathic arthritis: a preliminary study

**DOI:** 10.1186/s12969-018-0262-9

**Published:** 2018-07-04

**Authors:** Qianzi Zhao, Lawrence K. Jung

**Affiliations:** Division of Rheumatology, Children’s National Medical Center, 111 Michigan Ave, NW, Washington, DC 20010 USA

**Keywords:** B lymphocytes, Cytokines, Inflammation, Juvenile idiopathic arthritis, Synovial fluid

## Abstract

**Background:**

To understand the relationship between regulatory B cells (Bregs) and juvenile idiopathic arthritis (JIA), we analyzed the percentages of Bregs and their function in peripheral blood (PB) and synovial fluid (SF) of JIA patients.

**Methods:**

Twenty-one JIA patients and 11 children with growing pain but without known rheumatic diseases as controls were included. The B cell phenotype and intracellular production of IL-10 of Bregs were assessed by flow cytometry. Mononuclear cells from PB and SF were stimulated to produce IL-10 in vitro for the identification of IL-10- producing regulatory B cells.

**Results:**

The percentage of CD24^hi^CD38^hi^ Bregs in the PB of JIA patients was significantly decreased compared to that in controls, and it was even lower in the SF of JIA patients compared to that in the PB. CD24^hi^CD38^hi^ Bregs frequency was significantly lower in the PB of RF-positive patients than in RF-negative patients. Frequency of IL-10-producing regulatory B cells (B10 cells) was significantly lower in active JIA patients than that in inactive patients.

**Conclusions:**

The inability of the host to produce enough regulatory B cells in PB and especially in SF of JIA patients may contribute to the disease, especially the local inflammation.

## Background

Juvenile idiopathic arthritis (JIA) is the most common chronic rheumatic disease in children [1]. JIA is not one disease. Rather, International League of Associations for Rheumatology (ILAR) has classified it into 7 subtypes by the number of joints and the type of extra-articular involvement [2]. Children with JIA are at risk for joint damage, resulting in poor functional outcomes and decreased quality of life [3]. The pathogenesis of JIA is not known yet. Recent studies have suggested that B cells may have a role in these disorders. For example, B cell-related genes were up-regulated in JIA patients [4], and memory B cells were increased in oligoarticular and polyarticular JIA patients [5].

B cells are thought to play pathogenic role in the immune responses, due to their ability (a) to produce autoantibodies and (b) to act as antigen-presenting cells. However, evidences have accumulated showing that B cells can also down-regulate the immune response in both mouse and human [6–15]. Genetically B cell-deficient mice suffered more severe disease of experimental autoimmune encephalomyelitis [7]. When in vitro-activated B cells were transferred into mice in the collagen-induced arthritis mice model, they reduced the incidence and severity of disease [8, 13]. The term “regulatory B cells”, shorted as Bregs, was used to define the B-cell subset with regulatory properties [9].

There are several possible mechanisms by which B cells can regulate the immune responses [16–18]. Among these mechanisms, the ability to produce regulatory cytokine interleukin-10 (IL-10) is crucial in their regulatory function [8, 10, 12, 14, 16, 19–22]. Regulatory B cells that can produce IL-10 are termed as B10 cells. IL-10 is an anti-inflammatory cytokine that could regulate immune response by restoring Th1/Th2 balance and directly inhibit inflammatory cascade [23–25]. However, the ability of Bregs to suppress immune responses was not totally IL-10-dependent [10]. So B10 cells are a subgroup of regulatory B cells.

There is no unique surface marker to identify Bregs. CD19^+^CD24^hi^CD38^hi^ [10, 14, 26, 27] and CD19^+^CD5^+^CD1d^hi^ [28–31] have been used in different studies. It was reported that the majority of the CD19^+^CD5^+^CD1d^hi^ B cells were contained within the CD24^hi^CD38^hi^ B cell subset [14]. Therefore, we utilized CD19^+^CD24^hi^CD38^hi^ as a surface marker for Bregs in this study.

Deficiency of Bregs may lead to autoimmune diseases. Indeed, decreased Breg cells number or function have been identified in rheumatoid arthritis (RA) [32, 33], systemic lupus erythematosus (SLE) [10, 30], anti-neutrophil cytoplasmic antibodies (ANCA)-associated vasculitis [26, 34]. Transferred regulatory B cells could reduce disease activity in mouse arthritis model [8, 13]. Therefore, it is reasonable to hypothesize that Bregs may play a role in the pathogenesis of JIA. In this study, we test this hypothesis by analyzing the percentages of Bregs and their ability to produce IL-10 in peripheral bloods and synovial fluids of JIA patients.

## Methods

### Patients and controls

A total of 32 patients from the Division of Rheumatology of Children’s National Medical Center were recruited in this study, including 21 JIA patients (13 poly-JIA, 5 oligo-JIA, 2 systemic, 1 psoriatic) and 11 children with growing pain but no known rheumatic diseases as controls. JIA patients were diagnosed according to the ILAR criteria [2]. Growing pain was diagnosed after known diseases were excluded with negative immunologic test findings. Peripheral blood (PB) samples were collected from the JIA patients and controls. One patient was followed longitudinally and PB samples were collected at both active and inactive phase. Synovial fluid (SF) samples were collected from 4 JIA patients who required intra-articular steroid injection as a part of treatment protocol. Of these subjects, both PB and SF samples were collected from 1 patient on the same day. Disease activity was assessed and inactive disease was defined according to Wallace’s criteria [35]. Patients who didn’t meet the definition for inactive disease were defined as having active disease. Demographic and clinical data of the patients were collected. The study was conducted in compliance with the Helsinki Declaration and ethical approval was obtained from the Institution Review Board of Children’s National Medical Center (Pro00005055). All patients were enrolled after obtaining informed consent from parents and assent from patients older than 7 years old.

### Human cell isolation and generation of B10 cells

Peripheral blood mononuclear cells (PBMCs) and synovial fluid mononuclear cells (SFMC) were isolated from heparin-treated PB and SF by Ficoll-Paque Plus (GE Healthcare, Uppsala, Sweden) gradient centrifugation. PBMCs and SFMCs were cultured in RPMI 1640 containing L-glutamine (Life Technologies, Paisley, UK) supplemented with 100 U/μg/ml penicillin/streptomycin (Life Technologies, Paisley, UK), and 10% fetal bovine serum in 48-well flat-bottom plates for 48 h at 37 °C in 5% CO_2_. According to previous study [12], combination of CpG and CD40L stimulation could generate the most of B10 cells in human. Therefore, the cultured cells were stimulated with 10 μg/ml CpG ODN2006 (Invivogen, San Diego, USA) and 1 μg/ml CD40L (R&D Systems, Minneapolis, USA), or with phosphate-buffered saline (PBS) as control. For the last 6 h, 50 ng/ml phorbol myristate acetate (PMA) and 1 μg/ml ionomycin (Sigma-Aldrich, USA) were added to the stimulated cells; Brefeldin A (BFA, eBioscience, San Diego, USA), Golgi transport blocker, was added to all wells.

### Surface markers and intracellular IL-10 detection

The following anti-human monoclonal antibodies (mAbs) were used for surface markers and intracellular IL-10 detection: fluorescein isothiocyanate (FITC)-conjugated anti-CD19; phycoerythrin (PE)-conjugated anti-CD24 (BD Biosciences, San Diego, USA); PE-Cyanine7-congugated anti-CD38; allophycocyanin (APC)-conjugated anti-IL-10 (Biolegend, San Diego, USA); and isotype-matched and fluorochrome-matched control antibodies. Cells were stained with combinations of CD19-FITC, CD24-PE and CD38-PE-Cy7 mAbs for surface phenotype. For intracellular IL-10 detection, cultured cells were washed, fixed, permeabilized, and stained with IL-10-APC mAb. APC-conjugated isotype control was used for gate setting for cytokine expression. Stained cells were analyzed on an eight-color FACSCanto II flow cytometer (BD Biosciences) using FACSDiva software (BD Biosciences).

### Statistical analysis

Statistical analyses were performed using GraphPad Prism Version 6 (GraphPad Software, La Jolla, CA, USA). Chi-square tests were performed for discrete variables. Student *t* test was used for parametric test when comparing two groups with equal variances. Welch’s *t* test was used for parametric test when comparing two groups with unequal variances. Mann-Whitney *U*-test was used for non-parametric test when comparing two groups. Spearman’s correlation was performed to determine correlation. A *p* value of < 0.05 was considered statistically significant.

## Results

### Clinical characteristics of study subjects

The demographic and clinical features of 21 JIA patients and 11 controls are summarized in Table 1. There was no significant difference between the 2 groups with the exception that none of the control subjects were positive for rheumatoid factor. Table 2 shows the demographic and clinical features of 4 JIA patients from whom SF samples were collected.

**Table 1:** Demographic and clinical features of JIA patients and controls

Characteristics	JIA patients	Controls
Number	21^a^	11
Gender, Female:Male (n:n)	12:9	6:5
Age (years)	10.9 ± 1.1	12.4 ± 1.1
Duration of disease (years)	3.7 ± 0.7	NA
Treatment when sampled; n	NSAID, MTX, anti-TNF;4 NSAID, Anti-TNF; 3 MTX, anti-TNF;1 Anti-TNF;4 NSAID; 7 None; 2^b^	None; 5 NSAID alone;6
RF Pos:Neg (n)	4:14^c^	0:11
PB White blood cell count (× 10^9^/L)	7.62 ± 0.66	6.19 ± 0.54
PB Lymphocyte count (×10^9^/L)	3.06 ± 0.31	2.24 ± 0.12

^a^One patient was sampled at both active and inactive phase

^b^Patients were sampled when diagnosis was made and before treatment was given

^c^RF was not tested in 3 patients

*JIA* juvenile idiopathic arthritis, *MTX* methotrexate, *NA* not applicable, *Neg* negative, *NSAID* non-steroidal anti-inflammation drug, *Pos* positive, *RF* rheumatoid factor, *TNF* tumor necrosis factor, *PB* peripheral blood

**Table 2:** Demographic and clinical features of JIA patients from whom synovial fluid samples were collected

No.	Subtype	Gender	Age (years)	Duration of disease (years)	RF	Treatment when sampled
1	Oligoarticular	M	1.2	0.1	Neg	None^a^
2	Oligoarticular^b^	M	8	2.1	Neg	NSAID
3	Polyarticular	F	8	4	Neg	NSAID
4	Psoriatic	M	15	1	Neg	NSAID, MTX, anti-TNF

^a^This patient was sampled when diagnosis was made and before treatment was given

^b^Peripheral blood sample was collected from the patient the same day synovial fluid sample was collected

*F* female, *JIA* juvenile idiopathic arthritis, *M* male, *MTX* methotrexate, *NA* not applicable, *Neg* negative, *NSAID* non-steroidal anti-inflammation drug, *RF* rheumatoid factor, *TNF* tumor necrosis factor

### CD24hiCD38hi B cell levels were reduced in PB of JIA patients and even lower in SF

**Fig. 1:**
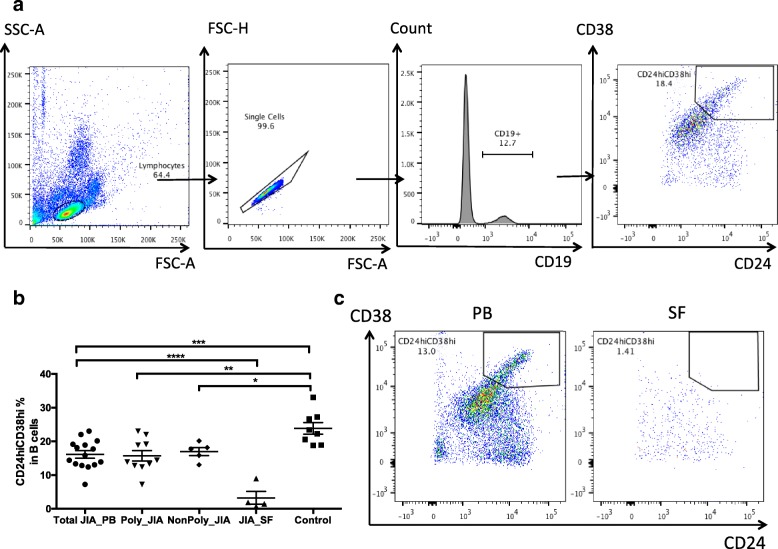
Frequencies of CD24^hi^CD38^hi^ Bregs in juvenile idiopathic arthritis (JIA) patients and controls. **a** The gate strategy for CD19^+^CD24^hi^CD38^hi^ cells in the peripheral blood (PB) of one control. B cells were defined as CD19^+^ lymphocytes. Within CD19^+^ B cells gate, CD24^hi^CD38^hi^ cells were defined as CD24^hi^CD38^hi^ Bregs. **b** CD24^hi^CD38^hi^ Bregs frequencies in total B cells were compared in PB of total, poly and non-poly JIA patients, synovial fluid (SF) of JIA patients, and PB of controls. The frequency of CD24^hi^CD38^hi^ Bregs in the PB of total JIA patients was significantly decreased compared to those in controls (*p* = 0.0007), and it was even much lower in the SF of JIA patients compared to that in the PB (*p* < 0.0001). **c** The percentage of CD24^hi^CD38^hi^ Bregs in the SF of one patient was lower than that in the PB of the same patient collected on the same day. *p < 0.05, **p < 0.01, ***p < 0.001, ****p < 0.0001

Peripheral blood and synovial fluid mononuclear cells from subjects were phenotypically analyzed by flow cytometry for their expression of CD19, CD24, and CD38 surface markers. B cells were defined as CD19^+^ lymphocytes. Within CD19^+^ B cells gate, CD24^hi^CD38^hi^ cells were defined as CD24^hi^CD38^hi^ Bregs. The gate strategy for CD19^+^CD24^hi^CD38^hi^ cells was illustrated by a representative staining of cells in a healthy control subject (Fig. 1a). The percentages of CD24^hi^CD38^hi^ Bregs subset were calculated as the ratios of gated targeted cells to total CD19^+^ B cells. The results were expressed as mean values ± standard deviation (SD).

As shown in Fig. 1b, we observed a significant decrease in the levels of CD24^hi^CD38^hi^ Bregs in the PB of JIA patients compared to those in controls (16.11 ± 1.09% vs. 23.83 ± 1.73%, *p* < 0.001). An even more significant decrease was seen in the SF of JIA patients compared to those in the PB (3.23 ± 1.92% vs. 16.11 ± 1.09%, *p* < 0.0001). The data of PB and SF samples from the same patient supported this result. The percentage of CD24^hi^CD38^hi^ Bregs in the SF was lower than that in the PB of the same patient collected on the same day (1.41% vs. 13.00%) (Fig. 1c). Of note, there was no significant difference of B cells percentages in PB between JIA patients and controls (15.22 ± 1.28% vs. 16.35 ± 1.22, *p* = 0.5788). However, B cells population in SF was significantly decreased compared with PB (1.47 ± 0.27% vs. 15.22 ± 1.28%, *p* = 0.0001). Probably due to this decrease, CD24^hi^CD38^hi^ Bregs subset was almost absent in SF mononuclear cells (SFMCs). In peripheral blood mononuclear cells (PBMCs) of JIA patients, the percentage of CD24^hi^CD38^hi^ Bregs was 2.41 ± 0.27%, while this percentage was 0.04 ± 0.02% in SFMCs, which was highly significant (*p* = 0.0004).

We next examined the frequencies of CD24^hi^CD38^hi^ Bregs in poly-JIA and non-poly-JIA patients. As in total JIA patients, the frequencies of CD24^hi^CD38^hi^ Bregs in poly-JIA and non-poly-JIA patients were significantly decreased compared with controls (*p* < 0.01 and *p* < 0.05, respectively) (Fig. 1b). No significant difference of frequency of CD24^hi^CD38^hi^ Bregs was observed between poly and non-poly JIA patients (*p* = 0.6177).

**Fig. 2:**
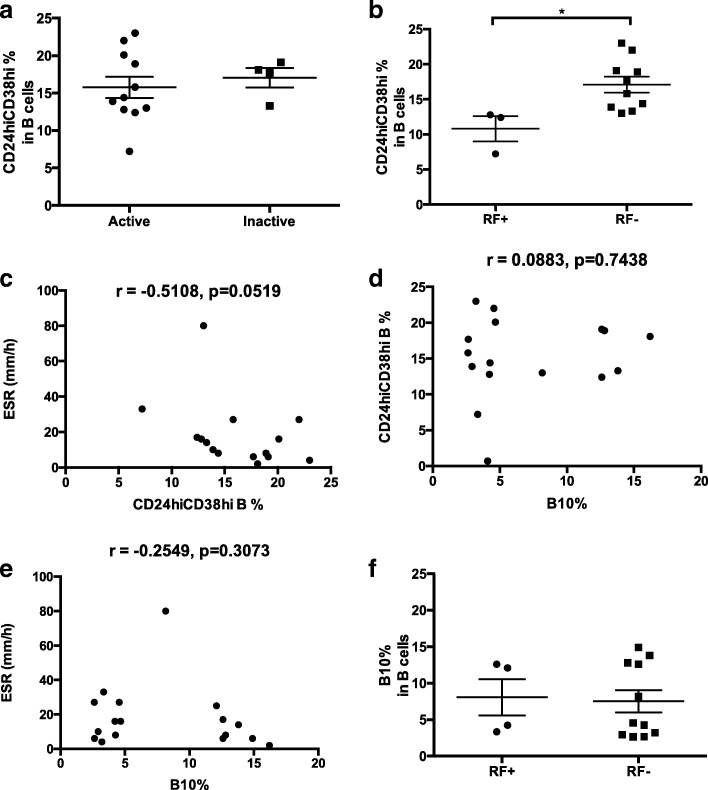
Correlation between frequencies of Bregs and different laboratory parameters. **a**. No difference of CD24^hi^CD38^hi^ Bregs frequencies was found between active and inactive JIA patients (8.07 ± 2.48% vs. 7.50 ± 1.52%, *p* = 0.8494). **b** The frequency of CD24^hi^CD38^hi^ Bregs was significantly lower in RF-positive JIA patients than in RF-negative patients (10.81 ± 1.80% vs. 17.11 ± 1.14%, *p* = 0.0199). **c** No significant correlation was found between frequencies of CD24^hi^CD38^hi^ Bregs and ESR (Spearman’s *r* = − 0.5108, *p* = 0.0519). **d** No significant correlation was found between frequencies of CD24^hi^CD38^hi^ Bregs and IL-10 producing regulatory B cells (B10 cells) (Spearman’s *r* = 0.0883, *p* = 0.7438). **e** No significant correlation was found between frequency of B10 cells and ESR (Spearman’s *r* = − 0.2549, *p* = 0.3073). **f** No difference of levels of B10 cells was found between RF-positive and RF-negative patients (8.07 ± 2.48% vs. 7.50 ± 1.52%, *p* = 0.8494). Bregs, regulatory B cells; ESR, erythrocyte sediment rate; JIA, juvenile idiopathic arthritis; RF, rheumatoid factor. * *p* < 0.05

Next, we examined whether CD24^hi^CD38^hi^ Bregs level correlated with disease activity or treatment. Treatments for all patients are shown in Table 1. As shown in Fig. 2a, there was no significant difference in the CD24^hi^CD38^hi^ Bregs levels between active JIA and inactive JIA patients (*p* = 0.6238). In addition, no difference of CD24^hi^CD38^hi^ Bregs level was observed between patients with and without methotrexate(MTX) or TNF antagonist treatment (*p* = 0.1358, *p* = 0.1469, respectively) (Table 3).

**Table 3:** Regulatory B cells percentages in different treatment subgroups

	No DMARD	DMARD	p value	No anti-TNF	Anti-TNF	p value
B cells (%)	14.96 ± 0.87 (*n* = 14)	16.19 ± 4.48 (*n* = 6)	0.7006	13.41 ± 1.07 (*n* = 8)	16.61 ± 2.20 (*n* = 12)	0.2757
CD24^hi^CD38^hi^ Bregs (% in B cells)	17.11 ± 1.12 (*n* = 11)	13.38 ± 2.43 (*n* = 4)	0.1358	18.08 ± 1.42 (*n* = 6)	14.80 ± 1.45 (*n* = 9)	0.1469
B10 cells (% in B cells)	7.25 ± 1.14 (*n* = 14)	7.73 ± 2.30 (*n* = 5)	0.8408	6.16 ± 1.48 (*n* = 7)	8.09 ± 1.34 (*n* = 12)	0.3683

*Bregs* regulatory B cells, *B10 cells* IL-10 producing regulatory B cells, *DMARD* disease-modifying antirheumatic drug, *JIA* juvenile idiopathic arthritis, *PB* peripheral blood, *SF* synovial fluid, *TNF* tumor necrosis factor

### CD24^hi^CD38^hi^ Breg cells levels were associated with RF in PB of JIA patients

We further examined the possible correlations between the frequencies of CD24^hi^CD38^hi^ Bregs and laboratory parameters. As shown in Fig. 2b, a significantly lower frequency of CD24^hi^CD38^hi^ Bregs was found in the PB of RF-positive patients than in RF-negative patients (10.81 ± 1.80% vs. 17.11 ± 1.14%, *p* < 0.05). However, no significant correlation was found between the frequencies of CD24^hi^CD38^hi^ Bregs and ESR (Spearman’s *r* = − 0.5108, *p* = 0.0519) (Fig. 2c).

### IL-10-producing regulatory B cells (B10 cells) percentages in JIA patients was increased in the poly-JIA patients although had no difference in JIA patients as a group

**Fig. 3:**
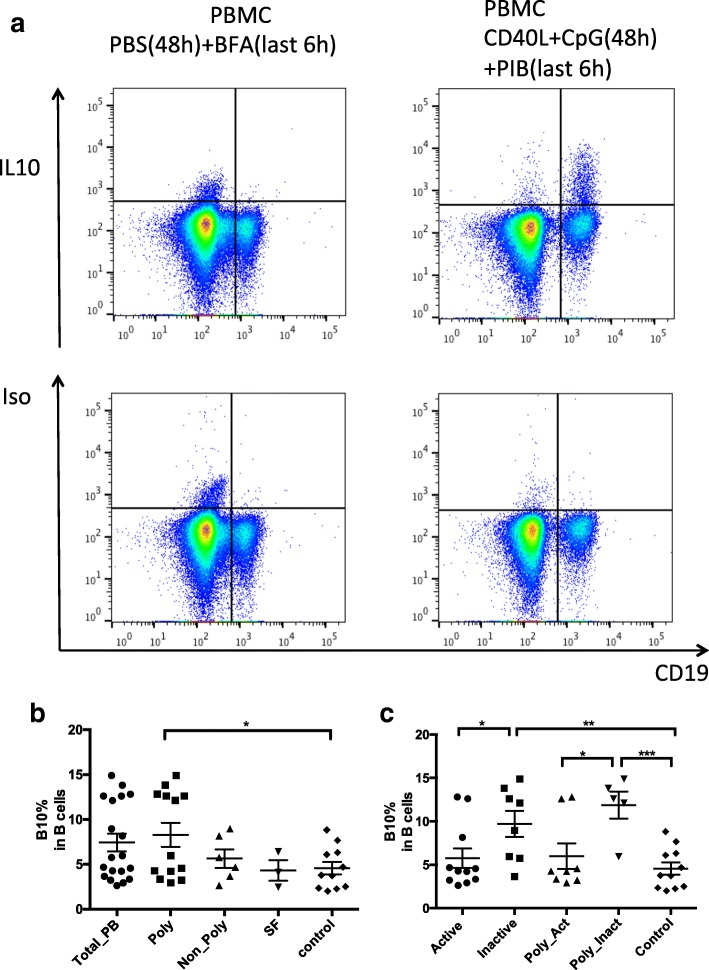
Frequencies of IL-10 producing regulatory B (B10) cells in juvenile idiopathic arthritis (JIA) patients and controls. **a** Representative intracellular IL-10 staining in B cells in peripheral blood (PB) of one JIA patient with/without stimulation of CpG + CD40L for 48 h and phorbol 12-myristate 13-acetate+ionomycin+brefeldin A (PIB) for the last 6 h (right). Few B cells produced IL-10 when cultured with PBS only (left). Isotype controls were used to set up the negative population (lower). **b** Comparison of B10 cells frequencies in PB of different groups of JIA patients, synovial fluid (SF) of JIA patients, and PB of controls (left). **c** Comparison of B10 cells frequencies in active and inactive patients. BFA, brefeldin A; CD40L, CD40 ligand; Iso, isotype; PBMC, peripheral blood mononuclear cells; PBS, phosphate-buffered saline; PIB, phorbol 12-myristate 13-acetate+ionomycin+brefeldin A; poly-JIA, polyarticular juvenile idiopathic arthritis. *p < 0.05, **p < 0.01,***p < 0.001

As a subgroup of Bregs, B10 cells level was also examined in PB and SF of JIA patients and PB of controls. By ex vivo stimulation of CpG + CD40L for 48 h and PMA + ionomycin for the last 6 h, intracellular production of IL-10 by CD19^+^ B cells was observed, while few B cells produced IL-10 when cultured with PBS only (Fig. 3a). A representative experiment showing intercellular IL-10 staining of B cells in a JIA patient is shown in Fig. 3a.

Unlike the remarkable reduction of CD24^hi^CD38^hi^ Bregs levels in JIA patients, there was no significant difference of B10 cells levels between the PB of JIA patients and controls (7.43 ± 0.99% vs. 4.56 ± 0.71%, *p* = 0.0527) (Fig. 3b). In SF of JIA patients the B10 cells level was not significantly different from that in PB (4.32 ± 1.14% vs. 7.43 ± 0.99%, *p* = 0.2419) either. However, when we examined B10 cells frequency in the poly-JIA subgroup, we noticed a significant increase compared with controls (*p* = 0.0249) (Fig. 3b).

### B10 cells frequency in JIA patients was associated with disease activity

We then examined whether the frequencies of B10 cells correlate with disease activity. Significantly lower frequency of B10 cells was found in active JIA patients compared with that in inactive patients (5.77 ± 1.13% vs. 9.72 ± 1.49%, *p* < 0.05), while no significant difference was found between active patients and controls (5.77 ± 1.13% vs. 4.56 ± 0.71%, *P* = 0.3762) (Fig. 3c). One patient was followed longitudinally and the same trend was noted: the percentage of B10 cells increased from 2.93% when active and increased to 5.96% when inactive, supporting the association between B10 level and JIA disease activity.

Same trend was also found in poly-JIA patient. As shown in Fig. 3c, when we subdivided poly-JIA patients into active and inactive subgroups, we observed a significantly lower frequency of B10 cells in the active poly-JIA subgroup compared with inactive poly-JIA subgroup (6.00 ± 1.48 vs. 11.87 ± 1.56, *p* = 0.0239), while no significant difference was found between B10 cells frequencies in the active poly-JIA subgroup and controls (*p* = 0.3531).

When we compared the frequencies of B10 cells with different treatment groups, no significant difference was observed between groups with and without MTX or TNF antagonist (*p* = 0.8408, *p* = 0.3683, respectively) (Table 3).

### There is no notable correlation between Bregs levels and B10 cells levels

Since B10 cells are a specific subgroup of Bregs, we examined whether there was a correlation between the level of CD24^hi^CD38^hi^ Bregs and the level of B10 cells in JIA patients. As shown in Fig. 2d, no notable correlation was observed (*p* = 0.7438). Also, there was no correlation between the level of B10 cells and ESR level (*p* = 0.3073) (Fig. 2e). No difference of levels of B10 cells was found between RF-positive and RF-negative patients (8.07 ± 2.48% vs. 7.50 ± 1.52%, *p* = 0.8494) (Fig. 2f).

## Discussion

In this study, we found that CD24^hi^CD38^hi^ Bregs percentage was remarkably lower in the peripheral blood of JIA patients compared with control, and it was even much lower in the SF of JIA patients. This decrease was seen in both the poly-JIA and nonPoly-JIA groups. Also, reduced PB CD24^hi^CD38^hi^ Bregs levels were associated with patients with positive RF. In contrast, B10 cells, a special subgroup of regulatory B cells that could produce IL-10, were not reduced in PB and SF of JIA patients compared with controls, but it was associated with disease activity. The B10 cells level was significantly lower in active JIA patients than in inactive patients; this finding is also true in the poly-JIA subgroup.

Peripheral blood CD24^hi^CD38^hi^ Bregs deficiencies have been described in several autoimmune diseases, including RA [32, 33], SLE [10, 30] and ANCA-associated vasculitis [26, 34]. In this study, we have not only showed a decreased level of CD24^hi^CD38^hi^ Bregs in the PB of JIA patients, but also the deficiency of these cells in the synovial fluid of JIA patients. In SFMCs, CD24^hi^CD38^hi^ Bregs subset was almost absent and was as low as 1.6% of that in the PBMCs.

Taken together, those findings suggest that CD24^hi^CD38^hi^ Bregs may be critical in controlling inflammation in JIA and the inability of the host to produce enough of them in PB and especially in SF may contribute to the disease. Moreover, even if the patients had inactive disease, the CD24^hi^CD38^hi^ Bregs level was still significantly lower than control, and there was no significant difference between active and inactive patients, suggesting that the reduced CD24^hi^CD38^hi^ Bregs was inherent in the JIA patients.

In addition, we noticed that in the RF-positive patients, the CD24^hi^CD38^hi^ Bregs level was lower than that in RF-negative patients.This suggests that Bregs may play a role in regulating the production of autoantibody such as RF. This concept is supported by a murine model of transplantation tolerance, wherein the production of alloantibodies was significantly reduced by adoptive transfer of Bregs [36].

It is interesting that despite the decrease of CD24^hi^CD38^hi^ Bregs, B10 cells levels were not decreased in PB and SF of JIA patients compared with controls, and there was no notable correlation between CD24^hi^CD38^hi^ Bregs levels and B10 cells levels in JIA patients. This result might suggest that although CD24^hi^CD38^hi^ Bregs were numerically deficient in JIA patients, their inherent ability to produce IL-10 was not compromised. This lack of correlation between phenotypically defined Breg cell subset and B10 cells has already been reported in adults [33, 34, 37]. The reason might be that B10 cells are not restricted to the CD24^hi^CD38^hi^ B cell subset, and other B cells subsets might contain more of them [33].

Interestingly, we observed an association of disease activity with B10 cells in JIA patients, although we didn’t observe a difference between the levels of B10 cells in PB of total JIA patients and controls. The B10 cells frequency was significantly lower in all active patients than in inactive patients. This was also true in poly-JIA subgroup of patients. The increase in the B10 cells in inactive patients may be indicative of a successful pathophysiological response to the inflammation. Kalampokis et al. [38] have recently reported the number of B10 cells in JIA patients and in health children. Similar to our result, they didn’t find a significant difference of B10 levels between JIA group and controls. However, they didn’t observe a significant difference of total IL-10-producing B cells levels between active and inactive patients. This discrepancy may be due to different definition for “inactive disease”. In our study, we used the more strict criteria to define inactive disease as an active joint count of 0, absence of uveitis and a PGA < 10 mm with normal ESR [35], while Kalampokis et al.*.* used only one criterion of PGA < 10 mm. Our result suggests that the status of disease activity is a very important consideration when one studies the B10 cells in JIA.

Our study showed that patients with active JIA had less B10 cells frequency compared with patients with inactive disease. This is consistent with the results in patients with RA [14, 33]. We didn’t find a correlation between CD24^hi^CD38^hi^ Bregs or B10 cells levels and MTX or TNF treatment in JIA patients. This is consistent with the result of Kalampokis et al.*.*. This result suggests that MTX or TNF treatment might not help to correct the altered immunological balance in JIA patients. Glaesener et al showed that the CD24^hi^CD38^hi^ transitional B cells was significantly decreased in patients receiving MTX compared with untreated patients [39]. In our study, we didn’t see a significant difference of CD24^hi^CD38^hi^ B cells percentages between patients with and without MTX treatment. The difference in our conclusion may be rooted in the fact that their study-subject composition was different from ours. In Glaesener et al’s study, the dominant group of patients was oligo-JIA patients (68%). In our study, poly-JIA patients were the dominant group (62%). Our poly-JIA patients required both MTX and anti-TNF treatment while none receiving only MTX; whereas in their study, the group receiving just MTX were predominantly oligo-JIA subjects (71%). Due to our small sample size, we could not perform detailed analysis of effect of MTX alone.

## Conclusions

- Regulatory B cells a relatively new area of research compared with regulatory T cells. In recent years, emerging data support their importance in diverse normal and pathologic processes.
- Our study showed for the first time that CD24^hi^CD38^hi^ Bregs percentage was remarkably lower in the peripheral blood of JIA patients compared with healthy control, and it was even lower in the synovial fluid of JIA patients.
- Also, the regulatory B10 cells level was inversely correlated with disease activity.
- Our study suggests that the inability of the host to produce enough regulatory B cells in PB and especially in SF of JIA patients may contribute to the local inflammation.
- These findings provide new insights in the pathogenesis of autoimmune diseases and may suggest a novel approach to control the disease processes.
- One caveat about our finding is that the numbers of SF samples and subgroup subjects were small in this preliminary study. Further studies with a larger sample size and more longitudinal follow up will be needed to confirm the findings reported here.

## Abbreviations

ANCA: Anti-neutrophil cytoplasmic antibodies
ANOVA: One-way analysis of variance
APC: Allophycocyanin
B10 cells: IL-10-producing regulatory B cells
BFA: Brefeldin A
Bregs: Regulatory B cells
ESR: Erythrocyte sediment rate
FITC: Fluorescein isothiocyanate
IL-10: Interleukin-10
ILAR: International League of Associations for Rheumatology
JIA: Juvenile idiopathic arthritis
mAbs: Monoclonal antibodies
MTX: Methotrexate
PBMCs: Peripheral blood mononuclear cells
PBS: Phosphate-buffered saline
PE: Phycoerythrin
PGA: Physician Global Assessment
PMA: Phorbol myristate acetate
Poly-JIA: Polyarticular juvenile idiopathic arthritis
RA: Rheumatoid arthritis
RF: Rheumatoid factor
SF: Synovial fluid
SLE: Systemic lupus erythematosus
TNF-α: Tumor Necrosis Factor-α
